# MicroRNA Dysregulation in Cystic Fibrosis

**DOI:** 10.1155/2015/529642

**Published:** 2015-06-22

**Authors:** Paul J. McKiernan, Catherine M. Greene

**Affiliations:** Department of Medicine, Royal College of Surgeons in Ireland, Education and Research Centre, Beaumont Hospital, Dublin, Ireland

## Abstract

The cystic fibrosis lung is a complex milieu comprising multiple factors that coordinate its physiology. MicroRNAs are regulatory factors involved in most biological processes and it is becoming increasingly clear that they play a key role in the development and manifestations of CF lung disease. These small noncoding RNAs act posttranscriptionally to inhibit protein production. Their involvement in the pathogenesis of CF lung disease stems from the fact that their expression is altered *in vivo* in the CF lung due to intrinsic and extrinsic factors; to date defective chloride ion conductance, endoplasmic reticulum stress, inflammation, and infection have been implicated in altering endogenous miRNA expression in this setting. Here, the current state-of-the-art and biological consequences of altered microRNA expression in cystic fibrosis are reviewed.

## 1. Introduction

Cystic fibrosis (CF) is a multifaceted autosomal recessive disease caused by mutations in the CF transmembrane conductance regulator (*CFTR*) gene. Although its pulmonary manifestations are responsible for the major morbidity and mortality associated with the disease, CF is also characterised by a multitude of clinical extrapulmonary manifestations. In addition, the great heterogeneity in disease severity among people with CF means that the design of therapeutic interventions is particularly challenging. Ultimately, a move toward personalised therapy will greatly enhance our treatment of CF. MicroRNAs (miRNA) are a class of regulatory biomolecules with important functions in numerous biological processes and are aberrantly expressed in many human diseases. Therefore, it is important to elucidate the roles of these molecules in CF pathophysiology.

## 2. MicroRNA

miRNAs are 20–25 nucleotide RNAs involved in the translational regulation of gene expression [1]. Although the term “microRNA” was first coined in 2001, the first miRNA,* lin-4*, was discovered eight years earlier by Lee and colleagues, in the nematode* Caenorhabditis elegans* [2]. Having been initially discovered to play important roles in developmental biology, interest in these small RNAs has dramatically increased since this time as they have been found to have significant roles in a range of other biological processes such as proliferation and apoptosis. The latest version of miRBase (http://www.mirbase.org/, v21 [3]), the most comprehensive microRNA bioinformatics repository, contains entries from 223 species corresponding to over 35,000 microRNAs. The database now contains over 2,000 human microRNA entries. Expression levels of miRNAs vary greatly between cells and tissues, and aberrant levels of miRNA are associated with many diseases in humans.

As a rule, mammalian miRNAs are initially transcribed in the nucleus into longer primary miRNA (“pri-mir”) of up to 1000 nucleotides in length. These stem-loop structured pri-mirs are generally transcribed by RNA Polymerase II and subsequently undergo cleavage in two sequential steps. The initial processing occurs in the nucleus by the RNA endonuclease (RNase) type III enzyme Drosha with the involvement of other proteins, as part of the “microprocessor complex.” Drosha cleaves the pri-mir liberating shorter hairpin pre-miRNA structures (“pre-mir”), which are approximately 70–100 nucleotides in length [4], and these are actively transported into the cytoplasm* via* a process involving the protein Exportin 5 [5]. Once in the cytoplasm, the pre-mir is further processed by the RNase III enzyme Dicer, resulting in a mature miRNA duplex with 5′ phosphate and two-nucleotide 3′ overhangs [6]. Duplexes consist of a mature miRNA “guide” strand and a “passenger” miRNA^*^ strand which, in general, is degraded.

Generally, microRNAs regulate gene expression posttranscriptionally by binding in a sequence-specific manner to miRNA responsive elements (MREs), particularly in the 3′ untranslated region (UTR), of a target mRNA. They are recruited by Argonaute (Ago) proteins, particularly Ago2 [7] to form the multiprotein RNA induced silencing complex (RISC) [8, 9]. miRNAs can guide the RISC to a target mRNA which then induces cleavage degradation or translational repression of that mRNA [10, 11]. Although most miRNA studies have largely focused on miRNA-mRNA interactions in the 3′UTR of target mRNA, these interactions can also occur in the 5′UTR and coding sequence (CDS) [12, 13].

As a single miRNA can regulate many target mRNAs and each mRNA may harbour several MREs, validation of targets can be difficult and time consuming. Since miRNA target interactions are complex, predictions are difficult. However, many computational tools are currently available for predictions and these are continuously improving. Peterson et al. [14] summarises the four approaches common to the target prediction tools currently used. These are the quality of seed match, evolutionary conservation of a particular microRNA, thermodynamics (specifically free energy) of miRNA::mRNA target binding, and site accessibility or mRNA secondary structure. Various online tools aid in these predictions and some well-known examples include TargetScan, PicTar, DIANA-microT, microrna.org, rna22, and RNAhybrid, which all utilise different algorithms and different sources of mRNA sequences. Yet, bioinformatic target prediction databases have high false positive and false negative rates, and experimental validation is ultimately required to truly determine miRNA::target mRNA binding and biological function.

It has been proposed that the expression and function of microRNAs themselves are regulated at three levels: transcription, processing, and subcellular localization [15]. At the level of transcription, miRNA expression can be controlled by many factors such as chromatin modifications, DNA methylation, and activity of transcription factors to name a few. miRNA processing can be affected by intrinsic or acquired alterations in the miRNA microprocessor machinery, thereby controlling miRNA function. A role for long noncoding RNA transcripts in the sequestration of miRNAs is emerging. These are termed “miRNA sponges,” given their ability to soak up miRNAs and reduce their interactions with target mRNAs. Additionally, single nucleotide polymorphisms (miRSNPs) that affect miRNA binding and function are being increasingly reported.

### 2.1. miRNAs in Lung Inflammation and Cystic Fibrosis

Analysis of multiple organs and tissues suggests that miRNAs have dual roles as both regulators of development and in maintenance of homeostasis [9, 16, 17]. Their importance in lung development is undisputed. Widespread changes in miRNA expression have been observed during lung development, and Dicer knockout mice, who have disrupted miRNA processing, display a lethal phenotype as a result of impaired lung growth [18]. Various studies demonstrate that miRNA expression remains relatively constant over time in the adult lung [19], supporting the notion that miRNAs play a central role in maintenance of lung homeostasis in the developed lung [16]. However, expression of miRNA is altered in pathological states, such as lung inflammation and disease. miRNAs have been shown to play important roles in the regulation of innate immunity and inflammation. At the most basic level, miRNAs are important in haematopoiesis and differentiation of immune cells [20, 21]. Numerous miRNAs are induced in innate immune cells, with miRs-155, -146, and -21 being expressed at particularly high levels [22, 23]. With known roles in regulation of inflammation, miRNAs are increasingly being examined within the context of inflammatory lung diseases such as CF.

**Figure 1:**
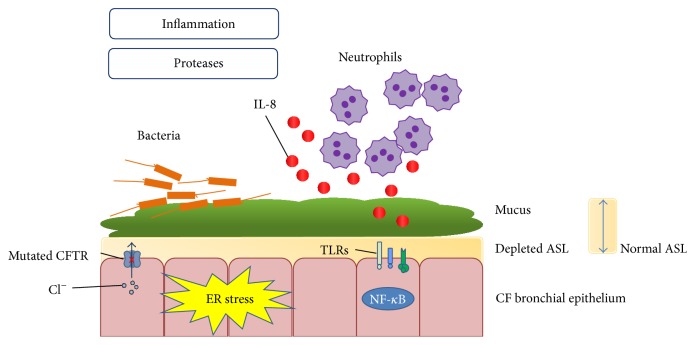
The CF airway lumen. Altered ion homeostasis in the CF airway due to mutated CFTR leads to impaired mucociliary clearance and a depleted ASL volume. This, coupled with intrinsic inflammation, leads to chronic bacterial infection and inflammation, with large numbers of neutrophils along with their secreted protease products being recruited to the lung. The high protease burden in the CF airway is damaging to lung tissue and leads to bronchiectasis and ultimately lung failure and death. IL-8: interleukin 8; ASL: airway surface liquid; TLRs: Toll-like receptors; NF-*κ*B: nuclear factor-*κ*B.

The CF airway lumen is a unique milieu (Figure 1). Lining the airway epithelium in the CF lung is a depleted airway surface liquid layer (ASL) and more mucus than normal. Impaired mucociliary clearance promotes bacterial colonisation and generates a highly proinflammatory environment wherein innate immune responses are frequently activated. Another characteristic of the CF airway lumen is the high numbers of infiltrating neutrophils which are inherently dysfunctional and contribute to the preexisting protease-antiprotease imbalance. Accumulation of misfolded CFTR may contribute to endoplasmic reticulum (ER) stress responses in the airway epithelium, and collectively these features are central to the pathology and physiology of CF lung disease. Our group was the first to examine miRNA expression in CF [24]. Numerous microRNAs had altered expression between CF and non-CF bronchial epithelium; the altered miRNAs were predicted to regulate expression of proteins involved innate immunity, inflammation, ion conductance, and ER stress, amongst others.

#### 2.1.1. Innate Immunity

The airway epithelium acts as an anatomical barrier to or primary defense against infection. These cells contribute to the barrier function* via* three essential components: intercellular tight and adherens junctions (regulating epithelial permeability), secreted antimicrobial factors, and the mucociliary escalator [25]. Furthermore, it acts as a key mediator of both innate and adaptive immune responses toward invading pathogens. Toll-like receptors (TLRs) are a key group of pattern recognition receptors which mediate the recognition of and response to microbial infections and are highly expressed on myeloid cells. The expression of TLRs is, however, not confined to immune cells, and these receptors are also expressed at high levels on other cell types, including airway epithelial cells (AECs) such as tracheal, bronchial, and alveolar type II cells. In the CF lung, TLRs expressed by AECs contribute to the airway immune response by regulating the expression and secretion of cytokines, chemokines, and antimicrobial peptides and through enhancing the expression of cell surface adhesion molecules [26].

Target of Myb1 (TOM1) is a Tollip-binding protein recently shown to act as a negative regulator of TLR2, TLR4, and IL-1*β* induced signalling pathways in CF bronchial epithelial cells [24]. TOM1 was predicted to be regulated by miR-126, a miRNA that is significantly downregulated in CF bronchial brushings compared to controls. To validate this observation the coexpression of miR-126 and TOM1 was evaluated in CF and non-CF bronchial epithelial samples and cell lines, and a reciprocal expression pattern was evident; the effect of overexpression of miR-126 on* TOM1* gene and protein levels was examined in a CF bronchial epithelial cell line, and a miR-126::TOM1 mRNA interaction was functionally validated using a reporter system. This was the first report of altered miRNA expression affecting innate immune responses in the CF lung and suggests that decreased miR-126 may engender a TLR hyporesponsive state which could be important at times of infective exacerbations where a rapid and robust response is required.

In addition to the epithelium, bone marrow derived cells such as monocytes, macrophages, neutrophils, and dendritic cells are important in the CF lung. These are constantly recruited to the infected CF lung to clear pulmonary pathogens, but numerous studies have suggested an impairment of these cells in the context of the CF. It has been well established that the CF lung is dominated by a neutrophilic inflammation. Although neutrophils are required for antimicrobial defense, their accumulation over periods of time and poorly controlled release of their toxic granular content can lead to parenchymal lung tissue damage [27, 28]. Neutrophils from people with CF have been found to release more elastase [29] and have defective phagocytic capacity and oxidative burst compared to controls [30]. Impaired bacterial killing by CF neutrophils has been shown to be a result of excessive protease cleavage of important molecules such as the IL-8 chemokine receptor CXCR1 on neutrophils [31] and also impaired CFTR-dependent phagosomal chlorination [32]. Recent work has shown that neutrophils from people with CF have altered cytosolic ion concentrations resulting in impaired degranulation [33].

Monocytes originate from precursors in the bone marrow and circulate in the bloodstream, until they are attracted to infection or inflammatory signals in particular tissues, such as the lung, where they differentiate into macrophage or dendritic cell populations [34]. The monocyte/macrophage lineage of myeloid cells has three primary roles in the immune response: phagocytosis, antigen presentation, and immunomodulation [35]. In the lungs, monocytes primarily differentiate into alveolar macrophages. These are excellent phagocytes, effective at rapidly clearing bacteria from the airways. Their numbers are increased in BALF of young noninfected CF patients [36] and similarly in CF mouse models [37, 38]. Emerging evidences suggests that these cells are hyperresponsive in people with CF, when exposed to bacterial agonists [39–41]. CF macrophages also appear to be defective in intracellular bacterial killing [42–44] and efferocytosis (i.e., scavenging of apoptotic neutrophils) [45–47]. Therefore myeloid cells play important roles in driving pathogenesis of the CF airways.

Hector and colleagues have examined miRNA expression in CF myeloid cells (neutrophils and mononuclear cells) and found changes in specific miRNAs including decreased miR-9 in CF neutrophils and increased miR-126 in CF mononuclear cells versus the same cells from healthy control cells (Andreas Hector, University of Tuebingen, personal communication). Functional studies will define if these changes in miRNA expression impact on dysfunctional processes such as those described above.

#### 2.1.2. Inflammation

The CF lung is a high protease milieu and bacterial-derived proteases can contribute to this protease burden. For example,* Pseudomonas aeruginosa* secretes the metalloproteases* Pseudomonas* elastase (PsE) and alkaline protease (APR), capable of cleaving a wide range of host proteins and of altering the physiology of the CF airways [48–50]. High numbers of neutrophils contribute significantly to the abnormally high concentrations of neutrophil-derived proteases, for example, neutrophil elastase [51–53], proteinase 3 [54], and cathepsin G [55]; however a range of other endogenously expressed cysteine, metallo-, and aspartyl proteases generated by other cell types are also important. These include the cysteinyl protease cathepsin S [56] which can be expressed by bronchial epithelial cells and antigen presenting cells such as macrophages and dendritic cells. Weldon and colleagues [56] have recently found that the expression and activity of cathepsin S is increased in the BALF of children with CF, including a cohort of* Ps. aeruginosa*-negative preschool children, compared to non-CF children with recurrent infection, indicating that upregulation of cathepsin S may be CF-specific. Interestingly, they illustrated that this is due, in part, to decreased miR-31 which they have shown regulates the transcription factor interferon regulatory factor 1 (IRF-1), which controls cathepsin S expression. Levels of miR-31 were lower in CF versus non-CF cell lines, primary bronchial epithelial cells, and bronchial brushings [57].

Other studies have looked at alternative roles of miRNA in other aspects of inflammation in CF. Infection with* Ps. aeruginosa* induces the production of proinflammatory cytokines such as IL-8 in the CF airway epithelium. Fabbri et al. [58] found that miR-93 is decreased in CF bronchial epithelial IB3-1 cells during infection with this CF pathogen. They also demonstrated that the decrease in miR-93 expression is correlated with an increase in IL-8 levels and that miR-93 directly targeted IL-8 mRNA.

#### 2.1.3. Ion Conductance

CFTR is the most important ion channel in CF. The* CFTR* gene encodes a membrane bound ion transport protein that belongs to the ATP-binding cassette (ABC) superfamily of transporter proteins [59]. The gene, containing 27 exons, was mapped by positional cloning in 1985 to the long arm of chromosome 7 (7q31) [60]. Its protein product, which is 1480 amino acids in length, primarily functions as an ion channel that, in concert with the Ca^2+^-activated Cl^−^ channel (CaCC) [61], works to secrete Cl^−^ and fluid required to hydrate the airway mucus but has the additional ability to transport bicarbonate [62] and glutathione [63]. Although the mechanism is still not fully understood, evidence is emerging for the role of CFTR in regulating the epithelial Na^+^ channel (ENaC) and the failure of mutated forms of CFTR in restricting salt absorption through ENaC [64]. Therefore, in the CF airway, epithelial CFTR dysfunction leads to airway surface liquid volume depletion due to an imbalance between CFTR-mediated Cl^−^ secretion and ENaC-mediated Na^+^ absorption [27]. Indeed transgenic mice overexpressing the *β*-subunit of ENaC develop a CF-like lung pathology [65] and have been used as a model of CF lung disease [66].

*CFTR* expression is a carefully controlled process that is spatially and temporally regulated. Transcription can begin at different start sites depending on the tissue or developmental stage in question. For example,* CFTR* is positively regulated by a selection of transcription factors including C/EBP proteins and FOXA factors, amongst others;* CFTR* is also posttranscriptionally regulated by miRNAs. A number of studies have examined the role of microRNAs in the control of CFTR expression and various microRNAs were demonstrated to regulate CFTR [67–75]. Although different experimental situations were examined, such as different cell lines and response to cigarette smoke, miR-101, miR-145, miR-494, and miR-509-3p have been repeatedly implicated in many of these studies, strongly highlighting their roles in regulating CFTR expression. For example, Oglesby and colleagues [71] demonstrated that miR-145, miR-223 and miR-494 were upregulated in CF bronchial brushings and cell lines, inversely correlated with CFTR levels, and were shown to directly target* CFTR* mRNA. The expression of these miRNAs also correlated with p.Phe508del mutation and* Ps. aeruginosa* colonisation. Ramachandran et al. [73] showed that miR-494 and miR-509-3p are increased in CF primary airway epithelial cells, regulate CFTR, and are regulated by NF-*κ*B. In the most recent study, Viart et al. identified miRNAs that participate in* CFTR* downregulation in the lung after birth [75]. Having compared the miRNA expression profiles of adult and foetal lungs, three miRNAs in particular (miR-145, miR-150, and miR-451) were found to have a temporal effect, being significantly upregulated in the adult lung and therefore contributing to downregulation of CFTR. They also demonstrated how inhibitors based on these miRNAs can affect* CFTR* gene expression and function in air-liquid interface culture and suggest that these may be developed as tools for CFTR correction in people with CF [76].

#### 2.1.4. ER Stress

The ER is the site of protein translation, folding, and processing for transport to secretory vesicles. Misfolded variants of CFTR, for example, the class II p.Phe508del-CFTR protein, accumulate in the ER and fail to reach the apical surface of epithelial cells to function as anion channels. ER perturbation can lead to ER stress and the initiation of signalling networks aimed at restoring ER equilibrium. One such network is the unfolded protein response (UPR). Recent evidence has implicated miRNAs in regulation of the UPR, in contexts other than CF [77–80]. However one recent study has examined whether altered miRNA expression regulates expression of UPR genes in CF airway epithelium [81]. Activating transcription factor 6 (ATF6) is an ER resident transcription factor and a key component of the UPR [82]. Its activation leads to transcriptional induction of ATF6-regulated genes which function primarily to restore correct protein folding in the ER.

The role of miRNA in basal regulation of ATF6 was investigated in CF and non-CF bronchial epithelial cells* in vitro* and* in vivo*. miRNAs predicted to target the 3′UTR of the ATF6 mRNA were identified. Three of these, miR-145, miR-221, and miR-494, were upregulated in a p.Phe508del-CFTR versus non-CF bronchial epithelial cell line and also in p.Phe508del-CFTR versus non-CF bronchial brushings. Expression of ATF6 was reciprocally decreased in CF both* in vivo* and* in vitro*. After experimentally validating ATF6 as a molecular target of these miRNAs through the use of a luciferase reporter vector containing the full length 3′UTR of ATF6, the human studies were complemented by analysing the expression of key miRNAs in a mouse model of CF lung disease. Expression of miR-221, which is also predicted to regulate murine ATF6, was significantly increased in native airway tissues of *β*ENaC-overexpressing transgenic mice with CF-like lung disease versus wild type littermates, demonstrating structural and functional conservation between humans and mice. These findings implicate *β*ENaC-overexpressing transgenic mice as a useful animal model for studies manipulating miR-221 levels* in vivo* using miRNA overexpression strategies to limit ER stress-mediated inflammation.

## 3. Concluding Remarks and Perspective

In this review, we have discussed current data regarding miRNA studies in cystic fibrosis. What is clear is that miRNA dysregulation exists in CF, with many studies highlighting an altered miRNA expression profile in the CF lung, be it in cell lines, primary cell cultures, or bronchial brush samples. Some of these aberrantly expressed miRNAs have been demonstrated to be involved in the regulation of key components of inflammatory signalling and, more recently, the UPR. Others have been shown to regulate the expression of CFTR itself. Such dysregulated miRNA may represent potential therapeutic targets. Although this is an emerging field, some work is beginning to be carried out with respect to the development of strategies to ultimately modulate miRNA levels* in vivo* in the CF lung, through the use of miRNA mimics and inhibitors [83]. Finally, the potential of miRNA as biomarkers of CF disease progression remains underexplored in comparison to other diseases such as cancers. The expression of these may become particularly useful for predicting and determining CF lung disease in infants and children, where currently used surrogate markers and biomarkers are of little use.
